# Protozoan Infections in Cancer Patients: A Case Series

**DOI:** 10.7759/cureus.65445

**Published:** 2024-07-26

**Authors:** Janice Kim, Tarek Zieneldien, John Greene

**Affiliations:** 1 College of Arts and Sciences, University of South Florida, Tampa, USA; 2 Internal Medicine, Moffitt Cancer Center, Tampa, USA

**Keywords:** protozoa and helminths, immunosuppression, entamoeba histolytica infection, giardia duodenalis, intestinal parasitic infections

## Abstract

Intestinal parasitic infections can cause significant morbidity and mortality in individuals with cancer. Despite this, they are often self-limiting in healthy individuals. *Entamoeba histolytica *is an anaerobic parasite that causes amebiasis in infected individuals. Poor sanitary conditions and endemic areas increase the risk of contracting amebiasis. Furthermore, giardiasis is a parasitic infection of the small intestine that is caused by *Giardia duodenalis*, a flagellated protozoan. In both cases, the disease burden is greater and the timeline is longer among immunosuppressed patients. Due to this, we aimed to more thoroughly characterize disease progression and treatment efficacy of these intestinal parasitic infections in cancer patients by presenting a case of intestinal amebiasis and enterocolitis due to *Entamoeba histolytica,* as well as two giardiasis cases, while also providing a review of the literature.

## Introduction

Intestinal parasitic infections are caused by parasites such as protozoa and soil-transmitted helminths that inhabit the intestines and are a significant threat to immunocompromised individuals, causing a plethora of clinical conditions [1]. Even though they seldom cause severe disease and are typically self-limiting in healthy individuals, cancer patients are particularly susceptible to opportunistic parasitic infections. Common microscopic intestinal protozoa causing intestinal parasitic infections include *Entamoeba (E.) histolytica*, *Giardia lamblia*, and *Cryptosporidium* spp. [1]. The outcomes and severity of parasitic infections are often influenced by innate and acquired host immunity. Furthermore, among immunosuppressed patients, the duration of illness and disease burden are also often longer and more severe. To illustrate, in individuals with hematologic malignancies, symptoms of secondary immunodeficiency include increased susceptibility to infections, frequent episodes of fever, and generalized weakness due to compromised immune systems from treatment side effects, creating an environment conducive to infections by enteric parasites such as *Cryptosporidium* spp. [2].

Amebiasis is a protozoan infection caused by *Entamoeba histolytica*. Any individual can be infected; however, it has a higher prevalence in countries with poor public health and low socioeconomic status due to poor sanitation, crowded living situations, and consumption of contaminated food or water. Individuals with weakened immune systems are at a heightened risk. Recent travelers to endemic areas are at a higher exposure risk. Transmission occurs through fecal-oral ingestion, poor hand hygiene, and water sources contaminated with fecal matter. The pathogenesis for the infection begins with the ingestion of cysts from contaminated sources, such as food or water, and transforms into trophozoites in the intestine [3]. The trophozoites can invade the intestinal wall resulting in colitis or the formation of liver abscesses and the trophozoites can exit the host via the feces and continue the cycle [3]. This infection occurs worldwide, about 40 million patients develop colitis and the occurrence of infection is most prevalent in Central and South America, Africa, and India [4]. Most *E. histolytica* infections are asymptomatic; however, the infection can progress and cause intestinal amebiasis. Gastrointestinal symptoms are gradual and include abdominal pain, diarrhea, bloody stools, and weight loss [3]. As symptoms tend to be nonspecific, the differential diagnosis is broad and can include diverticulitis, inflammatory bowel disease, and ischemic colitis. Risk factors that result in an increased severity of disease progression include pregnancy, malignancy, young age, and corticosteroid use. Additionally, individuals with compromised immune systems such as patients receiving immunosuppressive treatments are at increased susceptibility to this infection [5]. About 10% of *E. histolytica* infections progress and mostly cause gastrointestinal issues, however, it can also affect the liver as well [6]. If the pathogen reaches the mucosal barrier, it can travel to the liver and form amoebic liver abscesses. To diagnose amoebic infections, a stool polymerase chain reaction (PCR) is done, as it has the highest sensitivity in distinguishing *E. histolytica* infections [7]. Moreover, stool microscopy is an effective tool to diagnose amoebic infections. It utilizes either a saline mount, which helps detect motile trophozoites and cysts of parasites, and an iodine mount, which stains the cysts and trophozoites to make them more visible and easier to identify [8]. To treat the infection, metronidazole or tinidazole is used, followed by paromomycin or diloxanide furoate to eradicate cysts [9]. In liver abscesses, aspiration of the abscess may be necessary if it is not responding to antibiotics, is large, or is in danger of rupturing [9].

Furthermore, giardiasis is another parasitic infection, but it is caused by *Giardia (G.) duodenalis* (*Giardia lamblia*, *Giardia intestinalis*), a flagellated protozoan parasite that colonizes the lumen of the small intestine in vertebrate hosts [10]. In terms of Giardia infections, immunodeficiency, especially antibody deficiency, makes hosts susceptible to greater persistence and intensity of infection [10]. Individuals typically become infected by ingesting Giardia cysts present in contaminated water or food and other risk factors include poor hygiene, close contact with infected individuals, and traveling to endemic areas. The infection is often characterized by greasy stool, diarrhea, bloating, abdominal cramps, malabsorption, weight loss, and increasing fatigue [11]. In immunocompetent patients, self-limiting and asymptomatic infections are common. On the other hand, immunosuppression, particularly hypogammaglobulinemia, is a risk factor for the development of symptomatic *G. duodenalis* infection [12].

Hypogammaglobulinemia is a disorder characterized by low levels of serum immunoglobulins or antibodies [13]. In adults, common variable immunodeficiency (CVID) is the most common cause of hypogammaglobulinemia [13]. The majority of CVID patients experience an elevated susceptibility to pathogens that affect the mucous membranes of the upper and lower airways, as well as the gastrointestinal tract [14]. The usual treatment for giardiasis encompasses antibiotic therapy, with the first-line treatment typically being a nitroimidazole. Nonetheless, metronidazole has certain obstacles such as significant failure rates in eliminating protozoa from the intestine and concerns with patient compliance [11]. There have also been reports of increasing incidences of nitroimidazole-refractory infection, particularly among travelers from India and Asia [15]. Nitazoxanide, a thiazolide, is an antiparasitic agent that has been reported to be efficacious against a wide range of parasites, including *G. duodenalis* refractory to nitroimidazoles. Alternative treatments include the administration of mebendazole, paromomycin, and albendazole [11]. As of now, optimizing treatment strategies for giardiasis in cases of nitroimidazole-refractory *G. intestinalis* infection requires further research on drug resistance mechanisms and is an ongoing challenge that may necessitate combination therapies.

## Case presentation

Case 1

**Figure 1:**
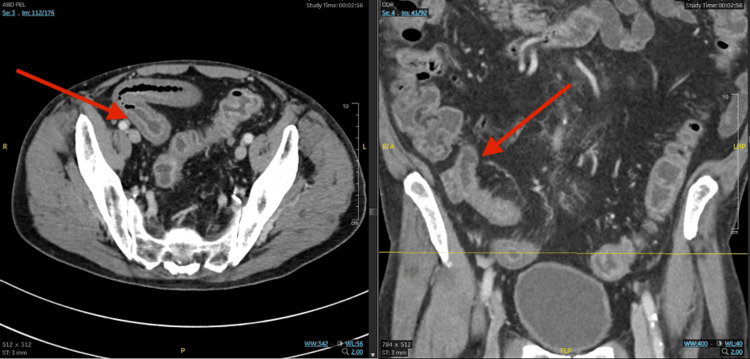
Coronal and sagittal abdominal CT scans in an amoebic colitis patient Diffuse inflammatory mural thickening of the terminal ileum and a diffuse fluid-filled colon are noted on the coronal and sagittal abdominal CT scans.

A 78-year-old male, who was diagnosed with esophageal cancer following a biopsy in December 2023 due to dysphagia, presented with progressive diarrhea that developed approximately two weeks after starting his treatment with FOLFOX (5-fluorouracil, oxaliplatin, and leucovorin), a chemotherapy regimen, on March 7, 2024. He experienced partial improvement while taking 2 mg of loperamide every 4 hours as needed with no more than 8 tablets in 24 hours and Lomotil; however, despite this, the diarrhea continued to worsen. An abdominal computed tomography (CT) scan revealed enterocolitis of the terminal ileum. Further testing through PCR tested negative for *Clostridium difficile* and through a gastrointestinal pathogen (GIP) panel, positive for *E. histolytica,* and the patient was admitted on April 3, 2024 (Table 1). On the day of presentation, diffuse inflammatory mural thickening of the terminal ileum and a diffuse fluid-filled colon were noted on the abdominal CT scans (Figure 1). Intestinal amebiasis and enterocolitis due to *E. histolytica* were diagnosed. Upon admission, he was found to have dyselectrolytemia and anemia (Table 2). He denied any fever, abdominal pain, recent traveling, eating outside his home, eating seafood or undercooked meat, having sick contacts, smoking, alcohol, or recreational drug use. However, he complained of multiple episodes of diarrhea daily. The patient was then started on 500 mg of metronidazole twice per day, by mouth for 10 days for intra-abdominal infection, followed by 250 mg of paromomycin three times a day by mouth for seven days to eliminate the intraluminal cysts.

**Table 1:** Pathogen test results in a patient with intestinal amebiasis GIP: gastrointestinal pathogen panel

Pathogen	Result
C. difficile PCR	Negative
GIP Campylobacter	Not Detected
GIP Plesiomonas shigelloides	Not Detected
GIP Salmonella	Not Detected
GIP Vibrio	Not Detected
GIP Vibrio cholerae	Not Detected
GIP Yersinia enterocolitica	Not Detected
GIP Enteroaggregative E. coli (EAEC)	Not Detected
GIP Enteropathogenic E. coli (EPEC)	Not Detected
GIP Enterotoxigenic E. coli (ETEC)	Not Detected
GIP Shiga-like toxin-producing E. coli	Not Detected
GIP Shigella/Enteroinvasive E. coli	Not Detected
GIP Cryptosporidium	Not Detected
GIP Cyclospora cayetanensis	Not Detected
GIP Entamoeba histolytica	Detected
GIP Giardia lamblia	Not Detected
GIP Adenovirus F. 40/41	Not Detected
GIP Astrovirus	Not Detected
GIP Norovirus GI/GII	Not Detected
GIP Rotavirus A	Not Detected
GIP Sapovirus	Not Detected

**Table 2:** Laboratory analyses during patient’s admission and follow-ups ALT: alanine transaminase; AST: aspartate aminotransferase

Lab Test	April 2, 2024	April 3, 2024	April 29, 2024	Reference Range
Sodium (mmol/L)	128	130 – 132	135	135 – 145
Potassium (mmol/L)	3.8	2.7 – 3.0	3.8	3.5 – 5.0
Hemoglobin (g/dL)	6.2 – 7.3	7.9 – 8.3	8.3	13.8 – 17.2
WBC (x10^3^/µL)	3.72	6.58 – 8.09	6.49	4.5 – 11.0
Platelet Count (x10^3^/µL)	84	92 – 104	183	150 – 400
Creatinine Level (mg/dL)	1.2	0.7 – 0.9	0.7	0.84 – 1.21
Total Bilirubin (mg/dL)	0.6	0.5 – 0.6	0.4	0.1 – 1.2
ALT (U/L)	26	23 – 25	20	7 – 56
AST (SGOT) (U/L)	34	35-36	40	10 - 40

Furthermore, on April 29, 2024, the patient was started on chemotherapy alongside external radiation therapy (XRT) with FOLFOX. On May 13, 2024, the patient presented back prior to week three of chemotherapy/XRT with FOLFOX, and he was tolerating it relatively well. He had continued fatigue, but his weight was mostly stable and he denied any fevers, chills, nausea, vomiting, abdominal pain, or diarrhea.

Case 2

**Figure 2:**
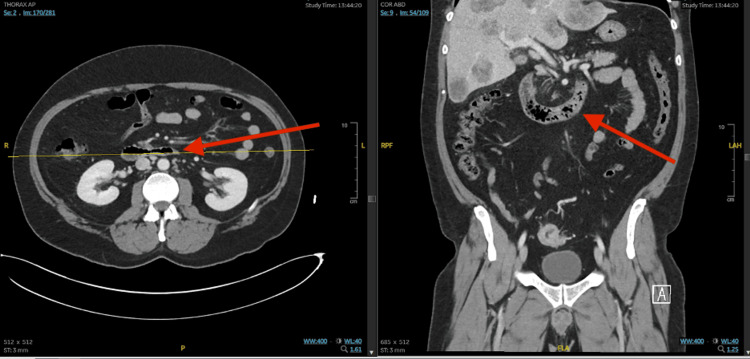
Axial and coronal abdominal CT scans on the day of presentation The CT scans show significant thickening of the small bowel wall in the jejunum.

A 43-year-old male with a recently diagnosed sigmoid colon mass and hepatic lesions suspicious for invasive adenocarcinoma presented on June 29, 2023, to the Moffitt Cancer Center for a liver biopsy, where he was found to be febrile and tachycardic. Thus, the procedure was canceled, and he was sent to urgent care. The patient reported that after eating Chinese food on June 26, 2023, he started having nausea, several episodes of vomiting, and a fever. He reported experiencing intermittent fevers since June 26, 2023, until admission, with a maximum temperature of 101 °F at home. Since then, he stated that he was not able to eat or drink much of anything, since each time he attempted to do so, he vomited. He admitted to loose stools that had been ongoing for months since he was diagnosed with a colon mass. The diarrhea has been unchanged from his baseline and he reported one to two episodes daily. The patient admitted to intermittent chronic gastrointestinal bleeding with small amounts of red blood in his stool. He also reported intermittent headaches secondary to excessive vomiting. The patient denied chest pain, shortness of breath, cough, sore throat, congestion, abdominal pain, sick contacts, or recent antibiotic use. The patient’s CT scans revealed considerable thickening of the small bowel wall in the jejunum (Figure 2).

In urgent care, his maximum temperature was 100.5 °F and he was tachycardic. A CT scan of the head revealed no acute abnormalities. A CT scan of the thorax, abdomen, and pelvis (CT TAP) also showed no acute findings to explain his symptoms. Laboratory results upon admission were significant for a white blood cell (WBC) count of 19.6x10^3^/µL, a procalcitonin level of 6.6 ng/mL, and a sodium level of 127 mmol/L. He was given a liter of normal saline in urgent care, started on maintenance intravenous fluids, and was admitted for further evaluation and treatment. On June 30, 2023, the GIP stool pathogen panel tested positive for enteroaggregative *Escherichia coli* (*E. coli*) and Giardia, which are likely the etiologies of his symptoms (Table 3). The patient was started on azithromycin for enteroaggregative *E. coli* and metronidazole for *Giardia lamblia*. After intravenous fluid resuscitation, the patient felt much improved. The headaches nearly resolved and no further episodes of nausea or vomiting were reported. The sodium level improved to 132 mmol/L and the WBC count decreased to 12x10^3^/µL (Table 4). Liver function tests also improved, with bilirubin levels remaining normal and aspartate aminotransferase mildly elevated at 46 U/L. The patient was discharged in stable condition and an order was placed to reschedule the liver biopsy.

**Table 3:** Pathogen screening test results GIP: gastrointestinal pathogen panel

Pathogen	Result
C. difficile PCR	Negative
GIP Campylobacter	Not Detected
GIP Plesiomonas shigelloides	Not Detected
GIP Salmonella	Not Detected
GIP Vibrio	Not Detected
GIP Vibrio cholerae	Not Detected
GIP Yersinia enterocolitica	Not Detected
GIP Enteroaggregative E. coli (EAEC)	Detected
GIP Enteropathogenic E. coli (EPEC)	Not Detected
GIP Enterotoxigenic E. coli (ETEC)	Not Detected
GIP Shiga-like toxin-producing E. coli	Not Detected
GIP Shigella/Enteroinvasive E. coli	Not Detected
GIP Cryptosporidium	Not Detected
GIP Cyclospora cayetanensis	Not Detected
GIP Entamoeba histolytica	Not Detected
GIP Giardia lamblia	Detected
GIP Adenovirus F. 40/41	Not Detected
GIP Astrovirus	Not Detected
GIP Norovirus GI/GII	Not Detected
GIP Rotavirus A	Not Detected
GIP Sapovirus	Not Detected

**Table 4:** Sequential laboratory analyses from admission to follow-ups ALT: alanine transaminase; AST: aspartate aminotransferase

Lab Test	June 29, 2023	June 30, 2023	July 25, 2023	Reference Range
Sodium (mmol/L)	127	132	140	135 – 145
Potassium (mmol/L)	4.0	3.8	3.7	3.5 – 5.0
Hemoglobin (g/dL)	12.6	10.6	12.3	13.8 – 17.2
WBC (x10^3^/µL)	19.67	12.98	8.33	4.5 – 11.0
Platelet Count (x10^3^/µL)	298-313	237	415	150 – 400
Creatinine Level (mg/dL)	0.8	0.8	0.8	0.84 – 1.21
Total Bilirubin (mg/dL)	1.5	0.9	0.3	0.1 – 1.2
ALT (U/L)	64	43	36	7 – 56
AST (SGOT) (U/L)	75	46	36	10 - 40

Case 3

A 64-year-old male with a medical history significant for multiple myeloma who had received conditioning chemotherapy with melphalan on January 16, 2023, and underwent an autologous bone marrow transplant infusion on January 18, 2023, presented with symptoms of neutropenic fevers on January 27, 2023. Afterward, he was admitted and denied feeling feverish or having any chills. He reported a cough over the previous three days, which had been productive of clear sputum. He also denied any chest pain, shortness of breath, nausea, vomiting, or abdominal pain. He has also been experiencing diarrhea over the last three days, with two episodes of watery bowel movements, one on the day prior to admission and one on the day after. He has been residing in a local hotel for the past 20 days before admission. Previously, he was living at his brother’s house. His brother had a poodle and a tropical bird, as well as chickens and goats, which he would occasionally feed, but he had no animal exposure since he arrived at the hotel. He denied contact with sick individuals but reported a sore throat and difficulty swallowing. 

The GI panel was positive for Giardia on January 27, 2023, and the patient was started on 500 mg of metronidazole by mouth, 2 mg of Imodium as needed for loose stools, and 1 tablet of Lomotil by mouth as needed for loose stools. The patient also received 2000 mg at 100 mL/hr of cefepime through IV piggyback for the neutropenic fevers and remained on 400 mg of fluconazole twice a day orally and 800 mg of acyclovir every 12 hours orally for neutropenic prophylaxis. After two seven-day courses of metronidazole, his previous diarrhea had completely resolved. He continued to take Lomotil one to two times per week with occasional soft stools, but he stated that his bowel movements had normalized and his energy levels had improved.

## Discussion

Parasitic infections encompass a wide range of diseases caused by various organisms that can cause infection and are broadly categorized as those caused by protozoa and helminths [16]. Protozoan infections often result from the ingestion of contaminated water or food and lead to severe gastrointestinal issues and, in some cases, life-threatening conditions, especially when immunocompromised patients are infected, resulting in a greater disease burden [17]. These patients have weakened immune systems, resulting in reduced numbers of immune cells, which leads to an inadequate immune response, thereby allowing pathogens to infect the patient and cause these life-threatening infections [18]. Additionally, atypical symptoms can appear, making it difficult to make an early diagnosis, increasing the risks of complications, and making treatment challenging [18]. Some protozoan infections include amebiasis, giardiasis, and malaria. Intestinal parasitic infections remain a major cause of morbidity and mortality worldwide. Despite risk factors for acquiring intestinal parasitic infections being similar in both immunocompetent and immunosuppressed patients, immunosuppression can affect the severity and timeline of the infection [12]. *E. histolytica* is an amoebic protozoan parasite and the infection can manifest in various forms, ranging from asymptomatic patients to severe infections, including liver abscesses and brain or lung infections [19]. These pathogenic parasites have three key virulence factors, which include Gal/GalNAc Lectin, which allows *E. histolytica* to adhere to host cells, amebapores to lyse host cells, and proteases to degrade host proteins [20]. On the other hand, giardiasis is caused by *G. duodenalis*, a flagellated protozoan parasite. *G. duodenalis* is a significant pathogen for immunodeficient patients or those with malnutrition. In this case series, we illustrate common risk factors associated with intestinal parasitic infections such as traveling to or living in locations of endemicity, malnutrition, or immunosuppression [21].

There are challenges in diagnosing diseases such as amoebic colitis in developed countries with low incidence. Misdiagnosis, significant variation in symptoms, and amebiasis masquerading as other conditions could potentially lead to late or missed diagnoses, which may cause severe, life-threatening complications [22]. Furthermore, treatments can also occasionally fail despite receiving successive courses. Regarding giardiasis, immunosuppression, inadequate drug levels, reinfection, although this is uncommon in developed countries where prevalence is low, and sequestration in the pancreatic ducts or gallbladder are some reasons [23]. Even more, immunosuppressed patients with common variable hypogammaglobulinemia or lymphoproliferative diseases involving the gastrointestinal tract are typically more susceptible to giardiasis, with their infections often being more difficult to cure [23]. To illustrate, even though patients with HIV infection and AIDS can typically be treated successfully, some do not respond to the typical course of therapies and develop life-threatening giardiasis [24]. These patients require specialized and comprehensive methods for treatment, which include highly active antiretroviral therapy, monitoring and follow-up, preventive measures, and also potential participation in clinical trials [25]. Due to these complexities, healthcare providers must maintain a high index of suspicion when encountering gastrointestinal symptoms, particularly in immunocompromised patients. These infections pose significant health risks, especially for immunocompromised patients undergoing cancer treatments that can impact treatment efficacy and patient well-being. By detailing treatment plans and addressing complications experienced by each patient, these cases can serve as a resource to guide future patient care strategies.

## Conclusions

Intestinal parasitic infections, such as amebiasis, can develop in patients who are under immunosuppressive conditions. The potential for prolonged illness and serious complications of these infections in symptomatic individuals presents significant challenges and raises the need for prompt diagnosis and initiation of effective treatments. There are many effective medications but the usual method is antiprotozoal agents followed by paromomycin to ensure all parasites are cleared to prevent relapse of the infection. Furthermore, for infection with *G. duodenalis*, standard treatments are typically curative, although some immunocompromised patients develop giardiasis that is refractory to the recommended treatment regimens. As such, combination therapies have been employed and deemed useful alternatives in cases of treatment failure. Nonetheless, further research and clinical trials are necessary to determine optimal treatment strategies for immunocompromised individuals with intestinal parasitic infections that are not responsive to standard courses of treatment. Overall, addressing the increasing healthcare concerns caused by intestinal parasitic infections in immunocompromised individuals is a difficult task, but it is necessary to prevent potentially life-threatening complications.
